# The Absence of *E. coli* Nucleoid‐Associated Protein FIS at Low Temperature Leads to an Adaptation Response That Causes a Shift Towards Genome Compaction in Small Rods

**DOI:** 10.1111/mmi.70082

**Published:** 2026-06-04

**Authors:** Pamela G. Jones

**Affiliations:** ^1^ Department of Biological Sciences Winston‐Salem State University Winston‐Salem North Carolina USA

**Keywords:** *Escherichia coli*, HsIVU protease, low temperature physiology, nucleoid condensation, nucleoid‐associated protein FIS, small rod morphology

## Abstract

In contrast to the rod shape at 37°C, the morphology of *Escherichia coli* cells at temperatures just above the minimum temperature of growth is small rods. A study was initiated to determine the requirement of nucleoid‐associated protein FIS for growth and genome compaction in the small rods at low temperature. Growth and nucleoid staining analyses revealed that the *fis* null mutant displayed decreased growth and initially formed filaments containing decondensed nucleoids at 12°C, indicating that FIS facilitates production of small rods with condensed nucleoids at low temperature. However, characterized by biphasic growth at low temperature, the *fis* null mutant exhibited increased growth, cell division, and nucleoid condensation following an acclimation phase. Therefore, the absence of FIS with nucleoid decondensation leads to an adaptation mechanism, termed FIS Null Adaptation Response, that causes a shift towards nucleoid condensation resulting in genome compaction in small rods. Furthermore, overproduction of the HsIVU protease suppressed the cold‐sensitive phenotypes of the *fis* null mutant indicating that degradation of a natural substrate of the protease alleviates the requirement of FIS at low temperature. In addition, null mutations of genes encoding natural substrates of HsIVU (exoribonuclease RNAse R, and cell division inhibitor SulA) were identified as extragenic suppressors of the *fis* null mutation.

## Graphical abstract

Unlike the wild‐type BW25113, the *fis* null mutant is characterized by biphasic growth at 12°C. During the first phase of growth, the absence of FIS results in the formation of filaments with dispersed nucleoids. During the acclimation phase, the FIS Null Adaptation Response is activated that leads to an increase in cell division and nucleoid condensation resulting in genome compaction in small rods during the second phase of growth.

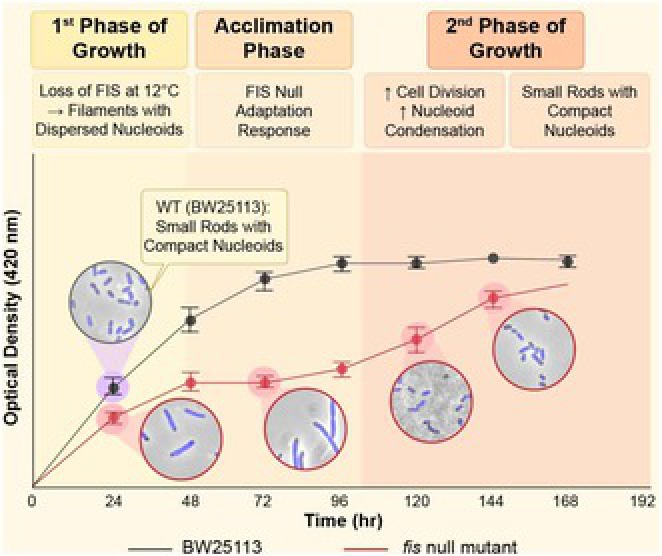

## 1 Introduction

Cellular stress occurs upon exposure of cells to an unfavorable environmental condition. Physiological changes are induced to counteract the damaging effects of the stress on cellular structures and processes. Among the physiological differences of cells growing exponentially at 37°C compared to cells growing exponentially at low temperature is a variation in morphology. While rods are formed at 37°C, small rods or coccobacilli are produced at temperatures just above 8°C, the minimum temperature of growth (Porter et al. 2016). The morphological transformation from rods at 37°C to small rods at low temperature is due to an increase in cell division, specifically requiring cell division proteins FtsN and DedD (Porter et al. 2016). Members of the SPOR domain family of *E. coli*, late cell division proteins FtsN and DedD bind to septal peptidoglycan stimulating septation (Gerding et al. 2009). FtsN is the only SPOR domain protein required for growth at 30°C and 37°C (Liu et al. 2019). Of the nonessential SPOR domain proteins (DedD, RlpA, and DamX), only DedD is specifically required to cause an increase in septation resulting in growth and the formation of the small rods at low temperature (Porter et al. 2016). Cells lacking either FtsN, even in the presence *ftsB* suppressor mutation that alleviates the growth and cell division requirement for FtsN at 30°C (Liu et al. 2019), or DedD displayed impaired growth and extensive filamentation at 10°C (Porter et al. 2016). The formation of the small rod cells is an adaptive response to low temperatures. Mutants that produced small cocci grew at a similar rate as the wild‐type strain at 10°C in LB media. In contrast, mutants that formed filaments had impaired growth at 10°C (Porter et al. 2016). Furthermore *E. coli* has been shown to produce small rods and coccoid cells in response to certain other stressful conditions, for example nutrient starvation, to conserve energy and to optimize the influx of nutrients and efflux of wastes in growth limiting environments (Young 2007).

The volume of ~1.6 mm *E. coli* chromosomal DNA must effectively reduce to sufficiently fit within the cell. In addition to macromolecular crowding and supercoiling, nucleoid‐associated proteins promote DNA condensation (Luijsterburg et al. 2006, 2008). Serving an architectural role on the chromatin, nucleoid‐associated proteins are small basic proteins that can wrap, bend or bridge distant DNA segments (Luijsterburg et al. 2006, 2008). One of several nucleoid‐associated proteins in *E. coli* , FIS can specifically recognize a 15‐bp motif as well as nonspecifically bind to DNA (Pan et al. 1998; Shao et al. 2008; Stella et al. 2010). Magnetic tweezers experiments showed that FIS binding to non‐specific sites resulted in significant DNA compaction due to DNA bending as well as formation and stabilization of DNA loops (Skoko et al. 2005, 2006). Contributing to DNA condensation, FIS introduces and constrains negative supercoiling (Schneider et al. 2001). Although the *fis* null mutant displayed a longer cell length and dispersed nucleoids at 30°C (Lioy et al. 2018), a specific growth requirement of FIS for global DNA compaction has not been clearly demonstrated. FIS is the most abundant chromatin protein during exponential growth (Talukder et al. 1999). In addition to shaping nucleoid structure, FIS acts as a transcriptional regulator of gene expression (González‐Gil et al. 1996; Kahramanoglou et al. 2011) and a regulator of DNA replication initiation (reviewed in Grimwade and Leonard 2021).

Because a role for FIS in cellular physiology at low temperature had not been investigated, a study was initiated to determine the requirement of FIS for growth, nucleoid condensation, and small rod morphology at temperatures near the minimum temperature of growth. In this work, the data indicate that FIS promotes growth and facilitates the organization of compacted nucleoids in the small rods. The *fis* null mutant exhibited biphasic growth accompanied by transient production of filaments containing large decondensed nucleoids at 12°C. Furthermore, the data demonstrate that *E. coli* responds to the lack of FIS by inducing an adaptation mechanism that leads to the formation of small rods with condensed nucleoids. Extragenic suppressor mutations of the *fis* null mutation are also identified that led to an increase in nucleoid condensation and/or cell division.

## 2 Results

### 2.1 FIS Facilitates Growth and Genome Compaction in Small Rods at Low Temperature

**FIGURE 1:**
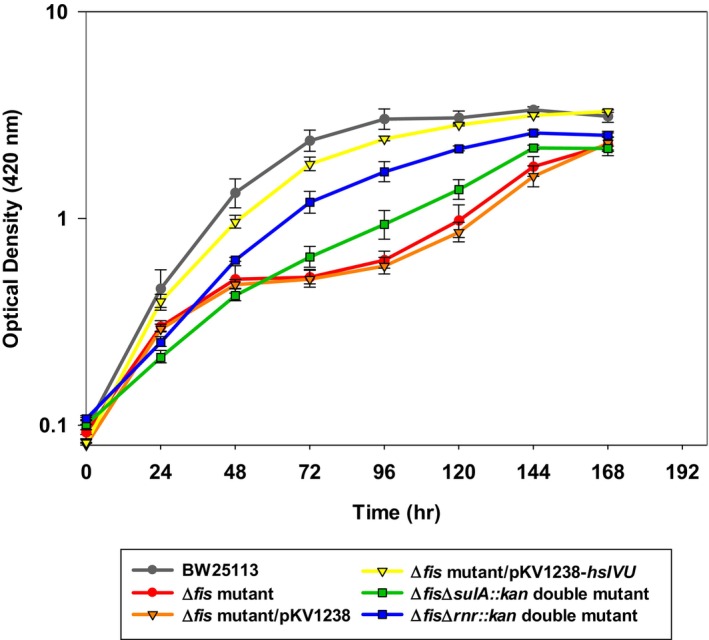
Effect of FIS on growth at 12°C. Growth of the wild‐type strain BW25113, *fis* null mutant, *fis* null mutant containing vector pKV1238, *fis* null mutant containing plasmid pKV1238‐*hsIVU* encoding two‐component protease HsIVU, Δ*fis*Δ*rnr::kan* double mutant, and Δ*fis*Δ*sulA::kan* double mutant at 12°C. Exponentially growing cultures in LB media at 37^ο^C were shifted to 12°C. After overnight incubation, the strains were diluted in LB media, and growth was monitored spectrophotometrically at optical density 420 nm for the times indicated. Data points are plotted with mean from at least three biological replicates. Error bars represent ± standard error of the mean (SEM).

Because cells lacking FIS grow similarly to the wild type but are elongated at 30°C (Lioy et al. 2018), experiments were done to determine whether FIS is required for growth and the small rod morphology at low temperature. Growth was monitored following dilution of an overnight culture in fresh LB media at 12°C. In comparison to the growth of the wild‐type strain BW25113, the growth of the *fis* null mutant was reduced at 12°C (Figure 1). Furthermore, exhibiting biphasic growth, the *fis* null mutant underwent an acclimation phase of ceased growth that began at 48 h and ended at 96 h. As a result, the first phase of growth extended from 0 to 48 h, and the second phase of growth began at 96 h. The biphasic growth of the *fis* null mutant indicates that *E. coli* acquired a mechanism during the acclimation phase that enabled cells to adapt to the absence of FIS at low temperature, resulting in the second phase of growth.

**FIGURE 2:**
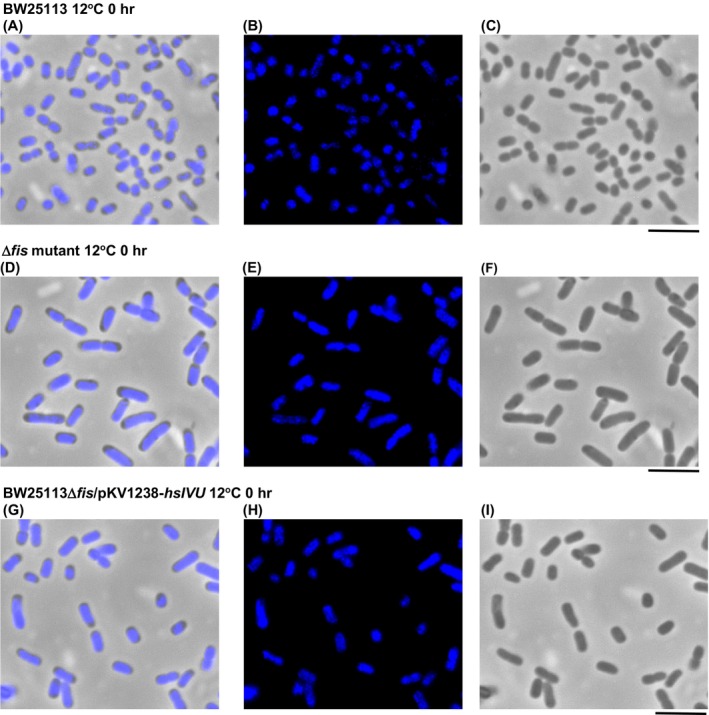
Effect of FIS on cellular and nucleoid morphology at 12°C at the 0 h. Phase‐contrast and fluorescent DAPI‐stained (false‐blue) overlay images (A, D, and G), fluorescent DAPI‐stained (false‐blue) images (B, E, and H), and phase‐contrast images (C, F, and I) of wild‐type strain BW25113, *fis* null mutant, and *fis* null mutant containing plasmid pKV1238‐*hsIVU* encoding two‐component protease HsIVU taken at a total magnification of 1000× (Bar = 5 μm).

**FIGURE 3:**
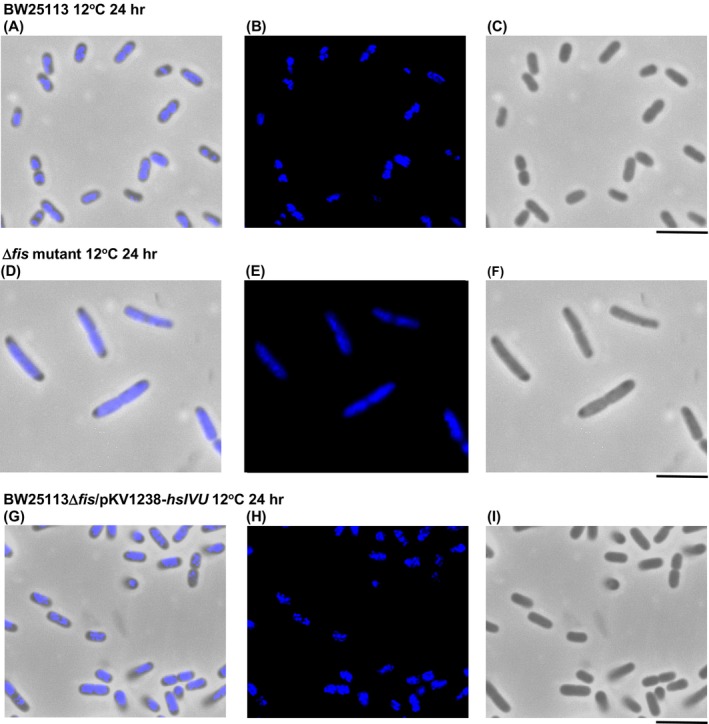
Effect of FIS on cellular and nucleoid morphology at 12°C at the 24 h. Phase‐contrast and fluorescent DAPI‐stained (false‐blue) overlay images (A, D, and G), fluorescent DAPI‐stained (false‐blue) images (B, E, and H), and phase‐contrast images (C, F, and I) of wild‐type strain BW25113, *fis* null mutant, and *fis* null mutant containing plasmid pKV1238‐*hsIVU* encoding two‐component protease HsIVU captured at a total magnification of 1000× (Bar = 5 μm).

**FIGURE 4:**
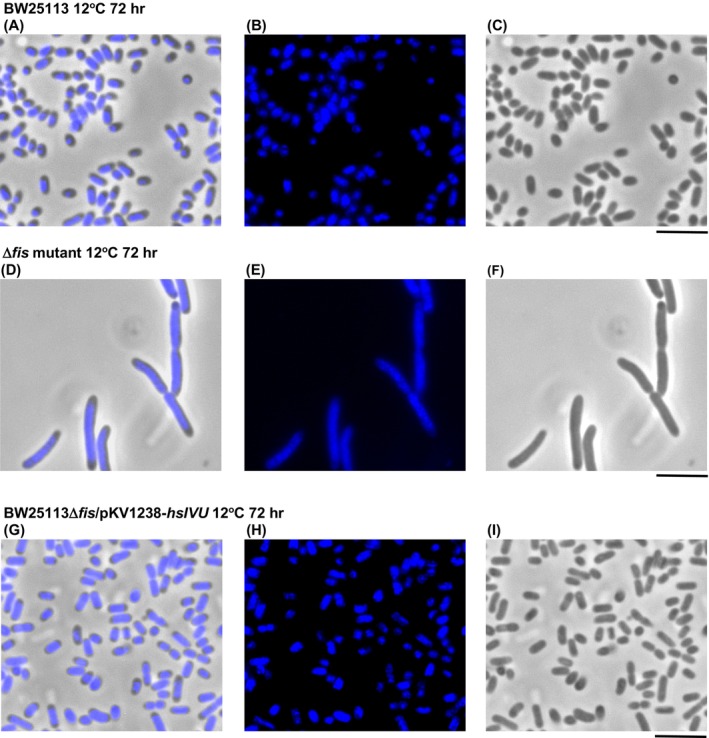
Effect of FIS on cellular and nucleoid morphology at 12°C at the 72 h. Phase‐contrast and fluorescent DAPI‐stained (false‐blue) overlay images (A, D, and G), fluorescent DAPI‐stained (false‐blue) images (B, E, and H), and phase‐contrast images (C, F, I) of wild‐type strain BW25113, *fis* null mutant, and *fis* null mutant containing plasmid pKV1238‐*hsIVU* encoding two‐component protease HsIVU taken at a total magnification of 1000× (Bar = 5 μm).

Microscopic examination of phase‐contrast and DAPI‐stained images showed that the wild‐type BW25113 cells at time zero were small rods packed with nucleoids (Figure 2A–C). The mean length of the cells was 1.17 μm (SD = ±0.22) and the mean width was 0.73 μm (SD = ±0.08) (Figure 8A,B). With a mean nucleoid length of 0.84 μm (SD = ±0.16), the mean nucleoid length to cell length (N/C) ratio was 0.72 (SD = ±0.05) (Figure 9A,B). By the 24 h, wild‐type BW25113 produced small rods, with a mean length of 1.48 μm (SD = ±0.34) and the mean width of 0.97 μm (SD = ±0.20), containing compacted nucleoids (Figures 3A–C and 8A,B). In comparison to the size of the wild‐type cells at time zero, the *fis* null mutant cells were larger (Figure 2D–F). The mean length and width of the *fis* null mutant rods were measured at 2.15 μm (SD = ±0.49) and 0.90 μm (SD = ±0.10), respectively (Figure 8A,B). The mean nucleoid length was 1.58 μm (SD = ±0.50) (Figure 9A). The mean N/C ratio was 0.73 (SD = ±0.05), which is similar to the ratio determined for the wild‐type cells (Figure 9B). In contrast to small rods with compacted nucleoids produced by the wild type at the 24 h, the *fis* null mutant formed filaments (Figure 3F) with a mean length of 5.11 μm (SD = ±1.23) and mean width of 1.14 μm (SD = ±0.22) during the first growth phase (Figure 8A,B). The nucleoids located in the *fis* null mutant cells were dispersed mainly throughout the filaments (Figure 3D–F). The mean nucleoid length was 4.40 μm (SD = ±0.99) compared to 0.98 μm (SD = ±0.23) of the wild‐type BW25113 (Figure 9A). The mean N/C ratio of the wild‐type BW25113 cells at the 24 h was 0.66 (SD = ±0.05) (Figure 9B). In comparison, the mean N/C ratio for the *fis* null mutant was 0.86 (SD = ±0.06) (Figure 9B), indicating that the nucleoid is decondensed compared to the nucleoid of the wild‐type BW25113 cells. Filaments with dispersed nucleoids were also formed by the *fis* null mutant at the 72 h during the acclimation phase (Figure 4D–F). For the wild‐type BW25113 at the 72 h, the mean length and mean width were 1.09 μm (SD = ±0.36) and 0.85 μm (SD = ±0.27); for the *fis* null mutant, 6.93 μm (SD = ±2.50) and 1.10 μm (SD = ±0.29) (Figure 8A,B). Furthermore at the 72 h, the mean nucleoid length for the wild type was 0.86 μm (SD = ±0.20) compared to 5.67 μm (SD = ±2.04) for the *fis* null mutant (Figure 9A). The data indicate that the function of FIS leads to an increase in nucleoid condensation and cell division resulting in optimal growth and small rods with compacted nucleoids at low temperature.

**FIGURE 5:**
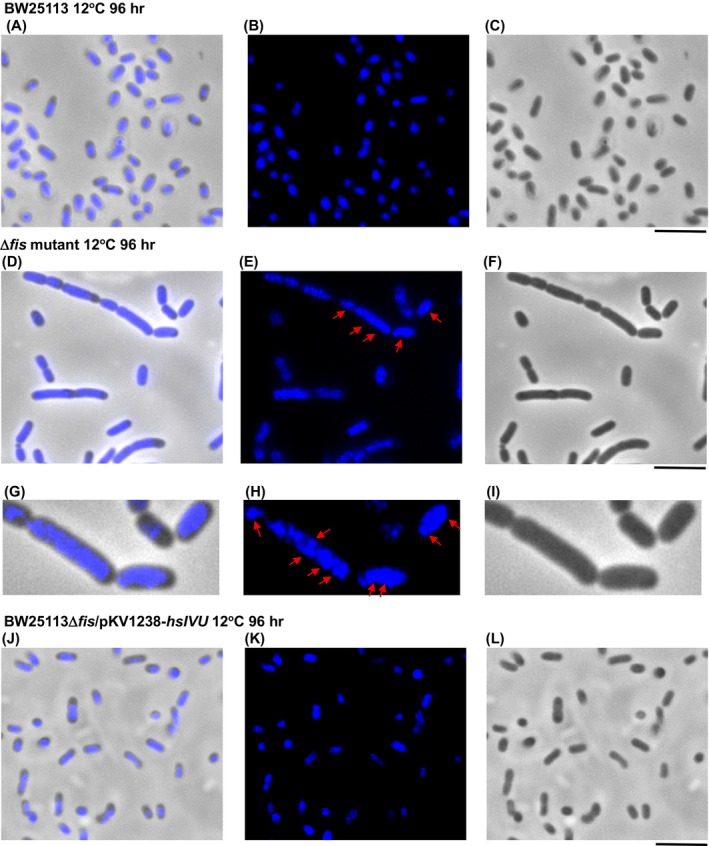
Effect of FIS on cellular and nucleoid morphology at 12°C at the 96 h. Phase‐contrast and fluorescent DAPI‐stained (false‐blue) overlay images (A, D, and J), fluorescent DAPI‐stained (false‐blue) images (B, E, and K), and phase‐contrast images (C, F, and L) of wild‐type strain BW25113, *fis* null mutant, and *fis* null mutant containing plasmid pKV1238‐*hsIVU* encoding two‐component protease HsIVU captured at a total magnification of 1000×. Enlarged images of *fis* null mutant cells with circular compacted nucleoids are shown in G–I. Red arrows (E and H) point to circular compacted nucleoids (Bar = 5 μm).

**FIGURE 6:**
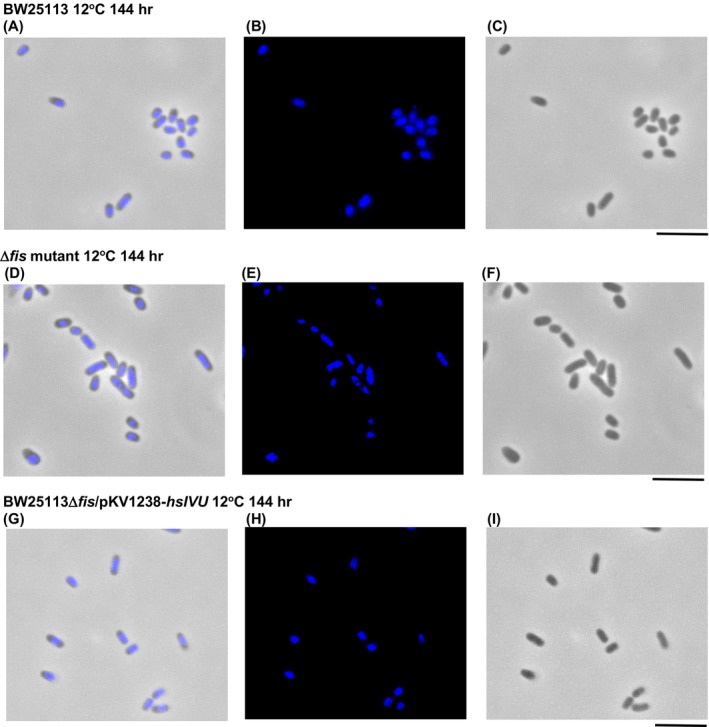
Effect of FIS on cellular and nucleoid morphology at 12°C at the 144 h. Phase‐contrast and fluorescent DAPI‐stained (false‐blue) overlay images (A, D, and G), fluorescent DAPI‐stained (false‐blue) images (B, E, and H), and phase‐contrast images (C, F, and I) of wild‐type strain BW25113, *fis* null mutant, and *fis* null mutant containing plasmid pKV1238‐*hsIVU* encoding two‐component protease HsIVU captured at a total magnification of 1000× (Bar = 5 μm).

Compared to the cell size at the 24 h, the size of the wild‐type BW25113 cells were smaller at the 72 h. At the 24 h, the mean length and width were 1.48 μm (SD = ±0.34) and 0.97 μm (SD = ±0.20) at the 72 h, 1.09 μm (SD = ±0.36) and 0.85 μm (SD = ±0.27) (Figure 8A,B). The nucleoid was also shorter at the 72 h. The mean nucleoid length was 0.86 μm (SD = ±0.20 μm) at the 72 h compared to 0.98 μm (SD = ±0.23) at the 24 h (Figure 9A). However, the mean N/C ratio for BW251123 was 0.79 (SD = ±0.06) at the 72 h compared to 0.66 (SD = ±0.05) at the 24 h (Figure 9B). It appears that the higher N/C ratio at the 72 h is mainly due to the decreased small cell size as a result of the wild‐type cells entering stationary phase (Figure 1). Compared to the 24 h, the compacted nucleoid occupied a larger percentage of the intracellular space of the smaller cell at the 72 h (Figure 4A–C) as well as cells during stationary phase at the 96 (Figure 5A–C) and 144 h (Figure 6A–C). This is further consistent with similar mean N/C ratios of the wild‐type BW25113 at the 96 h and 144 h, which were 0.80 (SD = ±0.06) and 0.77 (SD = ±0.05), respectively (Figure 9B).

### 2.2 Absence of FIS With Nucleoid Decondensation Leads to an Adaptation Response

At the onset of the second phase of growth at 96 h, the *fis* null mutant displayed filaments but also shorter filaments and rods that were formed as a result of an increase in cell division (Figure 5F). Cell constrictions leading to formation of shorter cells were also observed, which is consistent with the resumption of growth at the second phase. The mean length and width of the *fis* null mutant cells were measured at 2.63 μm (SD = ±1.67) and 1.01 μm (SD = ±0.21), respectively (Figure 8A,B). Furthermore, as shown in Figure 5D,E, there was an increase in nucleoid condensation. The microscopic images showed cells with circular compacted nucleoids, including some nucleoids that appear closely packed or merged together (indicated by the red arrows in Figure 5E). Enlarged images of the cells with the circular compacted and closely packed or merged nucleoids are shown in Figure 5G–I. Red arrows point to the condensed nucleoids in Figure 5H. The increase in nucleoid condensation is consistent with a decreased nucleoid length. The mean nucleoid length was 2.02 μm (SD = ±1.13) at the 96 h compared to 5.67 μm (SD = ±2.04) at the 72 h (Figure 9A). In addition, DAPI staining revealed an uneven distribution of the nucleoid at the 96 h (Figure 5D,E).

At the 144 h during the second phase of growth in the *fis* null mutant, there was a continued increase in cell division accompanied with genome compaction in the small rods (Figure 6D–F). At the 144 h, the mean cell length and width of the small rods were 1.58 μm (SD = ±0.60) and 0.89 μm (SD = ±0.13) (Figure 8A,B). In addition, the nucleoid of the *fis* null mutant cells was shorter and the mean N/C ratio was lower at the 144 h than at the 24, 72, and 96 h (Figure 9A,B). Specifically compared to 4.40 μm (SD = ±0.99) at the 24 h, the mean nucleoid length was 1.02 μm (SD = ±0.27) at the 144 h (Figure 9A). Compared to the N/C ratio 0.86 (SD = ±0.06) of the *fis* null mutant at 24 h, the ratio at 144 h was 0.65 (SD = ±0.05) (Figure 9B). The accumulated data indicate that the absence of FIS with nucleoid decondensation leads to an adaptation response that facilitates growth accompanied by increased cell division and genome compaction in small rods at low temperature.

### 2.3 *hslVU* Is a Multicopy Suppressor of the *fis* Null Mutation

Cell division protein FtsN is absolutely required to increase septation resulting in the formation of small rods at low temperature (Porter et al. 2016). Experiments were initially done to determine whether *ftsN* is a multicopy suppressor of the cold‐sensitive phenotypes of the *fis* null mutant. Plasmid pKD123 encodes FtsN (Dai et al. 1993). The presence of plasmid pKD123 in the *fis* null mutant resulted in increased growth at 10°C (data not shown). However, the presence of other FtsN‐encoding plasmids in the *fis* null mutant failed to restore the wild‐type growth (data not shown). Subsequent analysis of the plasmid pKD123 revealed that, in addition to gene *ftsN*, genes *hsIV* and *hslU* could also be expressed by the plasmid. Located adjacent to *ftsN*, genes *hslV* and *hslU* encoding HsIV (ClpQ) and HslU (ClpY), respectively, comprise the ATP‐dependent two‐component protease HslVU/ClpQY (Rohrwild et al. 1997). HslV (ClpQ) is the peptidase subunit, and HslU (ClpY) is the ATPase subunit. Experiments were conducted to determine whether *hslVU*, rather than *ftsN*, is the multicopy suppressor of the cold‐sensitive phenotypes of the *fis* null mutant. Plasmid pKV1238‐*hslVU* encodes the HslVU protease (Kanemori et al. 1999). Expression of the cloned *hslVU* operon is controlled from its native *hslVU* promoter. In comparison to the impaired growth of the *fis* null mutant with and without the vector pKV1238 at 12°C, the presence of plasmid pKV1238‐*hslVU* in the mutant resulted in growth as similarly observed as the wild‐type strain BW25113 (Figure 1). The presence of the plasmid pKV1238‐*hslVU* also resulted in the production of smaller cells even at 0 h (Figure 2F,I). Compared to the mean length of 2.15 μm (SD = ±0.49) and the mean width of 0.90 μm (SD = ±0.10) of the *fis* null mutant cells at the 0 h, the mean length and width of *fis* null mutant/pKV1238‐*hsIVU* cells were 1.44 μm (SD = ±0.35) and 0.84 μm (SD = ±0.10), respectively (Figure 8A,B). Furthermore, the presence of plasmid pKV1238‐*hsIVU* in the *fis* null mutant suppressed the formation of filaments at 24 (Figure 3I), 72 (Figure 4I), and 96 h (Figure 5L). At the 24 h for the *fis* null mutant/pKV1238‐*hsIVU* cells, the mean cell length was 1.68 μm (SD = ±0.38) and the mean cell width was 0.93 μm (SD = ±0.14) (Figure 8A,B). At the 72 h, the mean length and the cell width were 1.39 μm (SD = ±0.33) and 0.89 μm (SD = ±0.13); at the 96 h, 1.35 μm (SD = ±0.28) and 0.91 μm (SD = ±0.19) (Figure 8A,B). The presence of the plasmid pKV1238‐*hsIVU* also led to an approximately 80%–50% reduction in the nucleoid length (Figure 9A) resulting in the formation of small rods packed with nucleoids (as similarly observed for the wild‐type cells) at 24 (Figure 3G,H), 72 (Figure 4G,H), and 96 h (Figure 5J,K). Furthermore, the presence of the plasmid pKV1238‐*hsIVU* in the *fis* null mutant yielded a reduction of the mean N/C ratio from 0.86 (SD = ±0.06) to 0.76 (SD = ±0.05) at the 24 h and from 0.82 (SD = ±0.06) to 0.71 (SD = ±0.05) at the 72 h (Figure 9B). The data indicate that *hslVU i*s a multicopy suppressor of the impaired growth as well as the aberrant cellular and nucleoid morphology of the *fis* null mutant at low temperature.

The presence of the plasmid pKV1238‐*hsIVU* in the *fis* null mutant (compared to its absence) had a negligible effect on the mean N/C ratio at the 96 h, which was the start of the second growth phase of the *fis* null mutant (Figure 1). Although the plasmid pKV1238‐*hsIVU* resulted in a reduced mean cell and nucleoid length at the 96 h, the mean N/C ratio for the *fis* null mutant and *fis* null mutant with pKV1238‐*hsIVU* were similar, 0.77 (SD = ±0.05) and 0.75 (SD = ±0.05), respectively (Figure 9B). Furthermore, at the 144 h, the mean N/C ratio was lower for the *fis* null mutant compared to the *fis* null mutant/pKV1238‐*hsIVU*. For the *fis* null mutant, the mean N/C ratio was 0.65 (SD = ±0.05); for the *fis* null mutant/pKV1238‐*hsIVU*, 0.82 (SD = ±0.06) (Figure 9B). The lower mean N/C ratio for the *fis* null mutant is consistent with the increase in nucleoid condensation observed in the second phase of growth that resulted in genome compaction in the smaller cells at the 144 h (Figure 6D–F). The higher mean N/C ratio for the *fis* null mutant/pKV1238‐*hsIVU* (as similarly observed for the wild‐type BW25113) is also consistent with the nucleoid filling a larger percentage of the reduced cell volume during stationary phase at the 144 h (Figure 6G–I).

### 2.4 Null Mutations of *rnr* and *sulA* Are Extragenic Suppressors of *fis* Null Mutation

**FIGURE 7:**
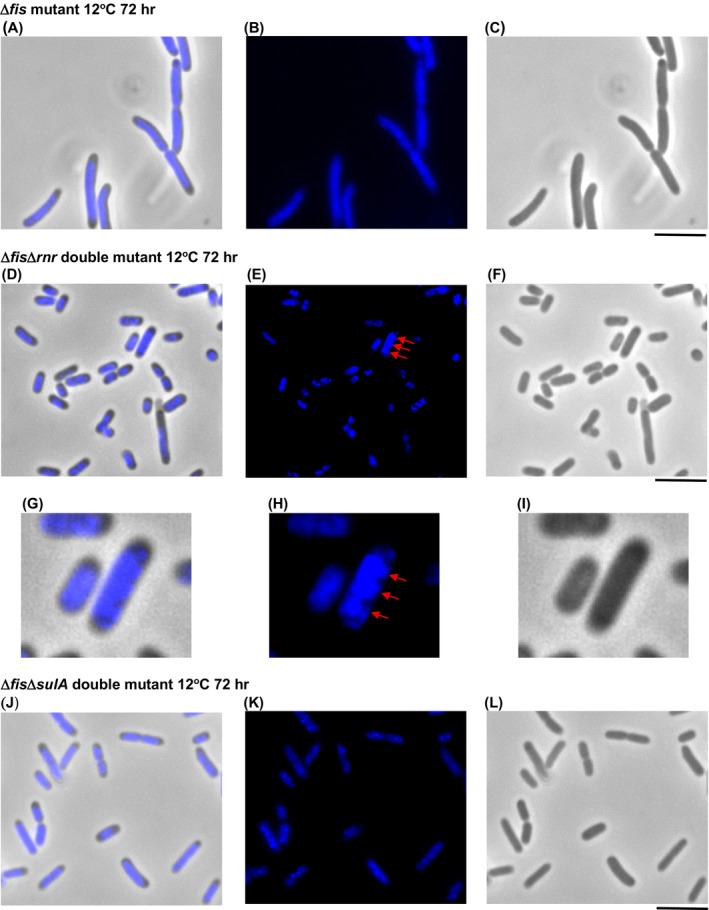
Extragenic suppression of aberrant nucleoid and cell morphology of the Δ*fis* mutant at 12°C at the 72 h. Phase‐contrast and fluorescent DAPI‐stained overlay images (A, D and J), fluorescent DAPI‐stained images (B, E and K), and phase‐contrast images (C, F and L) of the Δ*fis* mutant, Δ*fis*Δ*rnr::kan* double mutant, and Δ*fis*Δ*sulA::kan* double mutant captured at a total magnification of 1000×. Enlarged images of Δ*fis*Δ*rnr::kan* double mutant cells with circular compacted nucleoids are shown in G–I. Red arrows (E and H) point to circular compacted nucleoids (Bar = 5 μm).

**FIGURE 8:**
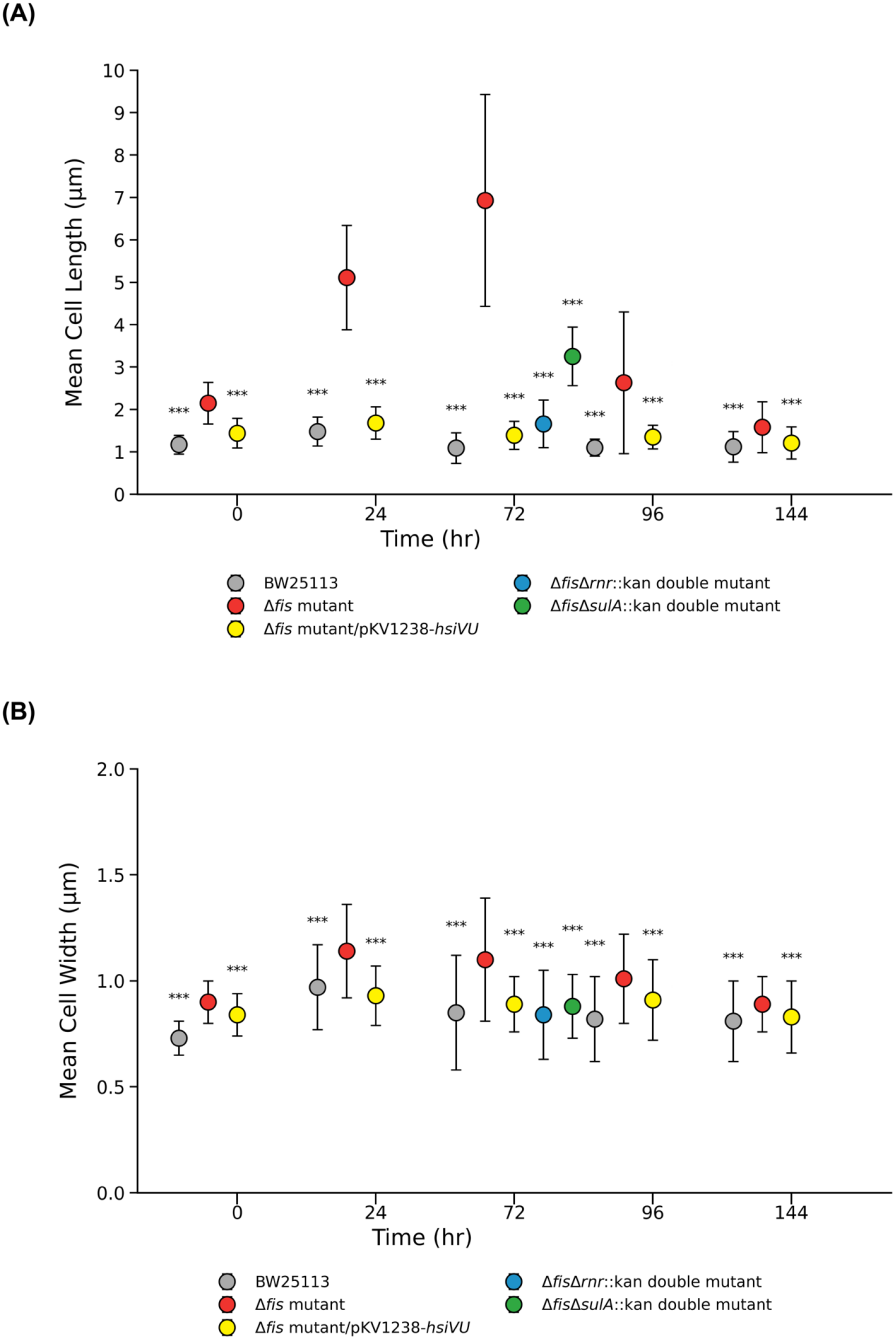
The effect of *fis* null mutation and extragenic suppressors on cell size. Mean cell length (A) and mean cell width (B) with ± standard deviation (SD) were determined at various times at 12°C from 3 biological replicates of at least 300 cells. One‐way analysis of variance for multiple comparisons was conducted with Dunn's post correction. The *fis* null mutant was compared to other strains at the corresponding time point. The *** indicates a statistical difference, *p*‐value < 0.001.

**FIGURE 9:**
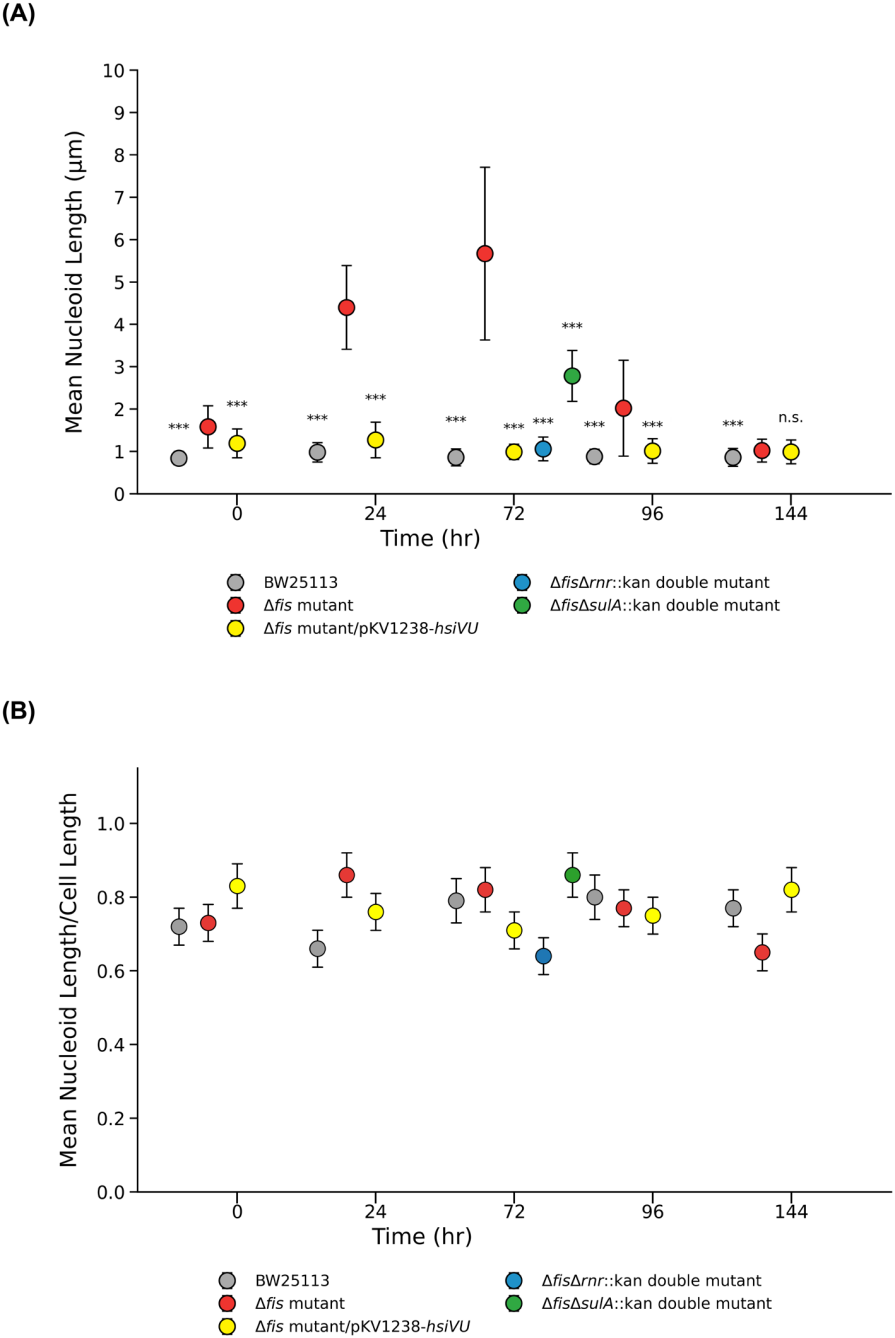
The effect of *fis* null mutation and extragenic suppressors on nucleoid length and nucleoid length‐to‐cell length (N/C) ratio. Mean nucleoid length (A) and mean nucleoid length‐to‐cell length (N/C) ratio (B) with ± standard deviation (SD) were determined at various times at 12°C from 3 biological replicates of at least 300 cells. One‐way analysis of variance for multiple comparisons was conducted with Dunn's post correction. The *fis* null mutant was compared to other strains at the corresponding time point. The *** indicates a statistical difference, *p*‐value < 0.001. n.s. indicates not statistically significant. Mean nucleoid length‐to‐cell length (N/C) ratio was calculated from the mean nucleoid length and mean cell length. To determine the standard deviation for the mean N/C ratio that resulted from mutational effects and/or environmental growth at 12°C, a relaxed coefficient of variation of 0.07 was applied (Chang et al. 2024; Gray et al. 2019).

The finding that *hslVU* is a multicopy suppressor of the cold‐sensitive phenotypes of the *fis* null mutant implies that specific destabilization of a substrate by the HsIVU protease leads to alleviation of the functional requirement of FIS at low temperature. Natural protein substrates of the HsIVU (ClpQY) protease include SOS‐induced cell division inhibitor SulA, capsule synthesis activation protein RcsA, 3′‐5′ exoribonuclease RNase R, and nucleoid‐associated protein YbaB (Tsai et al. 2017). Because growth studies demonstrated that SulA, RcsA, RNase R, and YbaB are not essential for growth at low temperature (data not shown), experiments were done to determine whether a null mutation of *sulA* or *rcsA* or *rnr* or *ybaB* is an extragenic suppressor of the *fis* null mutation at 12°C. Growth and microscopic analyses of wild‐type strain BW25113, Δ*fis* mutant, and the various double mutants were performed at 12°C. The Δ*fis*Δ*rcsA::kan* and Δ*fis*Δ*ybaA::kan* double mutants grew like the Δ*fis* mutant and formed filamentous rods (data not shown). Although the Δ*fis*Δ*rnr::kan* double mutant initially had slightly reduced growth compared to growth of Δ*fis* mutant at 24 h, the presence of the *rnr* null mutation led to suppression of the acclimation phase of ceased growth resulting in increased growth (Figure 1). The presence of the *rnr* null mutation also suppressed the aberrant filamentous and nucleoid morphology of the Δ*fis* mutant resulting in the production of small cells with compacted nucleoids at 72 h (Figure 7D–F). The mean length of the Δ*fis*Δ*rnr::kan* small cells was 1.66 μm (SD = ±0.56) and the mean width was 0.84 μm (SD = ±0.21) (Figure 8A,B). Additionally, at 72 h, the mean nucleoid length of the Δ*fis*Δ*rnr::kan* double mutant cells was 1.06 μm (SD = ±0.28) compared to 5.67 μm (SD = ±2.04) of the *fis* null mutant cells (Figure 9A). The mean N/C ratio of the Δ*fis*Δ*rnr::kan* double mutant was 0.64 (SD = ±0.05) compared to 0.82 (SD = ±0.06) of the *fis* null mutant (Figure 9B), further indicating that the presence of the *rnr* null mutation led to increased nucleoid condensation. This is consistent with the observation of a Δ*fis*Δ*rnr::kan* double mutant cell containing circular condensed nucleoids that appear merged together as indicated by the red arrows in Figure 7E. Enlarged images of the cells with the circular condensed nucleoids are shown in Figure 7G–I. Red arrows point to the nucleoids in Figure 7H. Therefore, the *rnr* null mutation is an extragenic suppressor of the cold‐sensitive phenotypes of the *fis* null mutant. In comparison, the *sulA* null mutation is a partial suppressor of the cold‐sensitive phenotypes of the *fis* null mutant. The deletion of the *sulA* gene from the *fis* null mutant led to moderately improved growth (Figure 1). In contrast to filaments produced by the *fis* null mutant, the presence of the *sulA* null mutation resulted in increased production of rods with a mean length and mean width of 3.25 μm (SD = 0.69) and 0.88 (SD = ±0.15) at 72 h (Figures 7L and 8A,B). The Δ*fis*Δ*sulA::kan* double mutant cells mainly contained dispersed nucleoids with a mean nucleoid length of 2.78 (SD = ±0.60) (Figures 7J,K and 9A). At 72 h, the mean N/C ratio was 0.86 (SD = ±0.06) (Figure 9B). In comparison, the mean N/C ratio of the *fis* null mutant was 0.82 (SD = ±0.06) (Figure 9B). Therefore, the presence of the *sulA* null mutation resulted in the formation of shorter cells but did not lead to increased nucleoid condensation.

## 3 Discussion

A DNA binding and bending protein, FIS serves an architectural role on the chromatin. Although in vitro studies showed that FIS DNA binding to nonspecific sites resulted in substantial compaction due to DNA bending as well as formation and stabilization of DNA loops (Skoko et al. 2005, 2006), an in vivo growth requirement of FIS for genome compaction has not been clearly demonstrated. In this work, the requisite of nucleoid‐associated protein FIS for growth, nucleoid condensation, and small rod morphology was examined at low temperature. Unlike growth and small rods with condensed nucleoids phenotypic of the wild‐type at 12°C, the *fis* null mutant exhibited reduced growth and initially produced filaments with decondensed nucleoids. The data indicate that FIS promotes growth and facilitates formation of small rods with compacted genomes at low temperature.

Biphasic growth typically describes the growth pattern of bacteria in response to the presence of two carbon sources. However, unlike at high temperature, the *fis* null mutant exhibited biphasic growth at low temperature. This finding indicates that the absence of FIS with nucleoid decondensation is a physiologically stressful condition at low temperature that leads to an adaptation mechanism termed FIS Null Adaptation Response. As a result, multiple and simultaneous cell divisions accompanied by nucleoid condensation occur leading to the production of small rods with compacted nucleoids at the second growth phase. Furthermore, there is a shift towards nucleoid condensation early in the second growth phase as evident by the formation of compacted circular nucleoids, some of which are merged or closely packed together. Condensed spherical and fused nucleoids are also induced by chloramphenicol as a result of an increase in nucleoid condensation (van Helvoort et al. 1996; Zusman et al. 1973). However, in contrast to the condensed nucleoids induced by chloramphenicol treatment, the compact nucleoids physiologically induced by the lack of FIS with nucleoid decondensation are part of an adaptation mechanism resulting in genome compaction in small rods at low temperature.

In *E. coli* , the morphology of cells growing at temperatures just above the minimum temperature of growth is small rods (Porter et al. 2016). Because the cells are small and space for the nucleoid is limited, the organization of highly condensed nucleoids is particularly advantageous at low temperature. Furthermore, it was reported that nucleoid compaction increases in response to a cold shock (Dash et al. 2022). Therefore, production of small rods with densely compacted nucleoids is beneficial for growth at low temperatures. This is also corroborated by the observation that expression of a mutant nucleoid‐associated protein HU in *E. coli* resulted in small coccoid cells with tightly condensed nucleoids accompanied by a markedly higher growth rate particularly at low temperatures (Kar et al. 2005). Therefore, FIS mediates an adaptive link between nucleoid condensation and small rod morphology facilitating nucleoid compaction in the small rods for growth at low temperature.

The finding that *hslVU* is a multicopy suppressor of the cold‐sensitive phenotypes of the *fis* null mutant indicates that destabilization of a substrate by the HsIVU protease leads to the alleviation of the requirement of FIS at low temperature. RNase R is a natural substrate of the HsIVU protease (Tsai et al. 2017). The data showed that a *rnr* null mutation is an extragenic suppressor of the abnormal growth as well as the aberrant nucleoid and cellular morphology of the *fis* null mutant at 12°C. This implies that proteolysis of RNase R by the HsIVU protease suppresses the functional requirement of FIS to facilitate genome compaction in the small rods. RNase R, a 3′‐5′ exoribonuclease, functions in the maturation and decay of highly structured RNAs (Andrade et al. 2009). A cold shock protein, RNase R also plays a role in regulating gene expression at low temperature (Cairrão et al. 2003; Phadtare 2012). In addition to the upregulation of several genes, DNA microarray analysis revealed that several genes are downregulated in the *rnr* null mutant, including *sulA* encoding the SOS‐induced cell division inhibitor (Mukherjee et al. 1998; Phadtare 2012). This suggests that the reduced expression of *sulA* contributes to the increase in cell division observed in the *ΔfisΔrnr::kan* double mutant. This is consistent with the finding that the cell division block in the *fis* null mutant is dependent upon SulA. In addition, genes encoding several membrane proteins are downregulated in the *rnr* null mutant at low temperature (Phadtare 2012). Transertion, which is coupled transcription and translation with translocation of nascent membrane proteins, is proposed to promote nucleoid expansion by facilitating attachment of the nucleoid to the membrane by transcription‐translation‐translocation chain (Woldringh et al. 1995; Woldringh 2002). Cumulative data from various studies indicate that chloramphenicol inhibits nucleoid expansion by causing membrane detachment of the nucleoid leading to nucleoid condensation and fusion (Bakshi et al. 2014, 2015; Spahn et al. 2025). The data showed that the presence of *rnr* null mutation in the *fis* null mutant resulted in nucleoid compaction. It is plausible that reduced co‐translation translocation of the membrane proteins (as a consequence of reduced expression due to the absence of RNaseR) leads to membrane detachment of the nucleoid resulting in nucleoid condensation in the *ΔfisΔrnr::kan* double mutant. This is supported by the observation of a *ΔfisΔrnr::kan* double mutant cell with circular compacted nucleoids. Furthermore, chloramphenicol treatment also induces the formation of spherical condensed nucleoids (van Helvoort et al. 1996; Zusman et al. 1973). The data suggest that the absence of RNase R in the *fis* null mutant effects gene expression, leading to a bypass of the requirement of FIS for formation of small rods with condensed nucleoids at low temperature.

Cell division inhibitor SulA, which is induced by DNA damage as part of the SOS response (Mukherjee et al. 1998), is a natural substrate of the HsIVU protease (Tsai et al. 2017). The data showed that a *sulA* null mutation is an extragenic suppressor of the filamentous phenotype of the *fis* null mutant at low temperature, resulting in the formation of rods. However, the *sulA* mutation did not restore nucleoid compaction in small rods, which is consistent with the partial growth restoration of the *fis* null mutant. The suppression of the filamentous phenotype as a result of the *sulA* deletion indicates that the absence of FIS at low temperature induces cell division inhibition by SulA. A generic response to stress, nucleoid compaction serves to insulate the DNA (Shechter et al. 2013). Under stressful conditions, an additional role for chromatin binding of nucleoid‐associated proteins is to avert damage (Hołówka and Zakrzewska‐Czerwińska 2020). The data indicate that the lack of FIS with nucleoid decompaction causes DNA to be susceptible to damage, triggering a SulA block in cell division. Therefore, FIS‐mediated nucleoid compaction aids in preserving genome integrity at low temperature.

## 4 Experimental Procedures

### 4.1 Bacterial Strains, Plasmids, and Growth Conditions

The bacterial strains utilized in this study were *E. coli* wild‐type strain BW25113 and the corresponding Keio deletion mutants JW3229 (Δ*fis*), JW5741 (Δ*rnr*), JW0941 (Δ*sulA*), JW1935 (Δ*rcsA*), and JW0460 (Δ*ybaB*) (Baba et al. 2006). From dilution of overnight cultures, growth of cultures in Luria‐Bertani (LB, Miller) media at 37°C was assayed spectrophotometrically at absorbance 420 nm. At an optical density 420 nm of 0.5 to 0.7, the cultures were shifted to 12°C followed by overnight incubation. After dilution of the cultures in fresh LB media, growth at low temperature was monitored at optical density 420 nm. When provided, ampicillin at 50 μg/mL or kanamycin at 30 μg/mL was administered to the cultures. Plasmid pKD123 encoding cell division protein FtsN (Dai et al. 1993) as well as vector pKV1238 and plasmid pKV1238‐*hsIVU* encoding two component protease HsIVU (Kanemori et al. 1999) were utilized in this study.

### 4.2 Construction of Double Null Mutants

As a result of transformation into *fis::kan* mutant strain JW3229, plasmid pCP20 (Cherepanov and Wackernagel 1995) containing a temperature‐sensitive origin of replication was used to remove the kanamycin resistance gene generating an ampicillin sensitive and kanamycin sensitive *fis* deletion strain. P1 transduction was used to transfer the *rnr::kan* mutation in JW5741, *sulA::kan* mutation in JW0941, *rcsA::kan* mutation in JW1935, or *ybaB::kan* mutation in JW0460 into the kanamycin sensitive *fis* deletion mutant. The double mutants were isolated by growth on LB plates containing kanamycin at 37°C. Polymerase chain reactions were done to confirm the presence of the null mutations.

### 4.3 Microscopy, Staining, & Cell Dimension Measurements

Ethanol‐fixed cells were applied to poly‐L‐lysine coated slides and were stained with DAPI (4′,6‐diamidino‐2‐phenylindole) in slow fade gold antifade reagent (Invitrogen) as described (Usongo et al. 2008). Stained cells were viewed with Olympus BX43 equipped with 100 W mercury lamp and DAPI filters under a total magnification of 1000×. Phase contrast optical system of Olympus BX43 was used to view cells under a total magnification of 1000×. Photographs were taken with a DP23M digital microscope camera, and images were analyzed using Cellsens software version 4.2. Phase contrast images were used for cell measurements. Fluorescent DAPI‐stained (false‐blue) images were used for nucleoid measurements. For each time point, mean dimensions (length and width) with standard deviation were obtained from at least 300 cells of three biological replicates. To determine statistical difference, one‐way ANOVA followed by Dunn's multiple comparison testing was performed using SigmaPlot.

## Author Contributions

**Pamela G. Jones:** conceptualization, investigation, funding acquisition, writing – original draft, writing – review and editing, methodology, formal analysis, project administration, resources, supervision, data curation, visualization, validation.

## Funding

This work was supported by the National Science Foundation (Grant 2101124).

## Ethics Statement

This is not a clinical study. Approval was not required.

## Conflicts of Interest

The author declares no conflicts of interest.

## Data Availability

The data that support the findings of this study are available from the corresponding author upon reasonable request.
